# Occurrence of tissue cyst forming coccidia in Magellanic penguins (*Spheniscus magellanicus*) rescued on the coast of Brazil

**DOI:** 10.1371/journal.pone.0209007

**Published:** 2018-12-18

**Authors:** Igor Cunha Lima Acosta, Rodrigo Martins Soares, Luis Felipe Silva Pereira Mayorga, Bruna Farias Alves, Herbert Sousa Soares, Solange Maria Gennari

**Affiliations:** 1 Departamento de Medicina Veterinária Preventiva e Saúde Animal, Faculdade de Medicina Veterinária e Zootecnia, Universidade de São Paulo–USP, São Paulo, SP, Brasil; 2 Instituto de Pesquisa e Reabilitação de Animais Marinhos—IPRAM, Cariacica, ES, Brasil; 3 Mestrado em Medicina e Bem estar animal, Universidade Santo Amaro, Av. Prof. Eneas de Siqueira Neto, São Paulo, Brazil; NIH, UNITED STATES

## Abstract

The main motivation for this study was to determine the occurrence of *Toxoplasma gondii*, a cosmopolitan widespread zoonotic parasite distribution that can infect a wide variety of mammals and birds, in Magellanic penguins (*Spheniscus magellanicus*) in Brazil. In recent decades there has been a significant increase in the number of penguins originating from Argentinian and Chilean Patagonia, where these birds are born, that arrive on the Brazilian coast, where many of them are stranded and rescued. Tissue samples were collected from 330 individuals surveyed from 2012–2015 at the Institute for Marine Animal Research and Rehabilitation (IPRAM) located in Cariacica, state of Espirito Santo, Brazil. Serum were collected from 145 animals surveyed in 2015 for the detection of anti-*T*. *gondii* antibodies using the Modified Agglutination Test (MAT ≥20) and 18 birds were positive, with titers of 20 (7 birds), 40 (9 birds) and 80 (2 birds). Mouse bioassay for the isolation of *T*. *gondii* was performed using tissues from 54 penguins that were also surveyed in 2015, but no isolates were obtained. DNA from tissue samples of 330 individuals was PCR amplified and sequenced to detect tissue cyst forming coccidians by using pan sarcocystids-directed primers (based on 18S rDNA). These samples were from animals surveyed in 2015 and from frozen stocked tissues from animals surveyed in the years 2012 and 2013. The positives were PCR amplified and sequenced with genus Sarcocystis-specific primers (based on internal transcribed spacer 1, RNA polymerase beta subunit coding gene, and cytochrome B coding gene) and with *Sarcocystis falcatula*/*Sarcocystis neurona*- specific primers (based on surface antigens SAG2, SAG3 and SAG4). Sixteen (3.0%) of pectoral muscle samples were positive by all the seven molecular markers and all the samples were identical to each other. Organisms close related to *Sarcocystis falcatula* were confirmed in all cases. This is the first report on molecular detection of infection by *S*. *falcatula*-related organisms and the first report of seropositivity for *T*. *gondii* in free-living Magellanic penguins in Brazil. Felids and didephid opossums are definitive hosts of *T*. *gondii* and *S*. *falcatula*, respectively. Where the penguins acquire the infective forms of the parasites shed by the terrestrial mammals remains to be elucidated.

## Introduction

Tissue cyst forming coccidian is a group of organisms comprising protozoa that form tissue cysts in intermediate hosts. This group can be divided into three subfamilies: Sarcocystinae, represented by the genera *Sarcocystis*; Cystoisosporinae, containing the genus *Cystoisospora*, and Toxoplasmatinae, with the genera *Toxoplasma*, *Neospora*, *Hammondia* and *Besnoitia* [1,2].

*Toxoplasma gondii* is an obligate intracellular parasite that develops asexually in the tissues of intermediate hosts (mammals and birds), infecting even humans. Its definitive hosts, feline, are the only ones able to eliminate oocysts in the environment through their feces [2]. Studies about the occurrence of anti-*T*. *gondii* antibodies in wild birds in Brazil have already been carried out on animals of various orders [3–7].

The genus *Sarcocystis* comprises more than 196 valid species that are differentiated by their morphological, biological and molecular characteristics. Tissue cysts are found in muscle and central nervous system of homeothermic and poikilothermic animals, i.e., mammals, birds and reptiles; the complete cycle of only 26 of these species is known [1]. *Sarcocystis falcatula*, which stands out among the *Sarcocystis* species pathogenic to birds, causes a severe respiratory disease. The disease has been described in captive psittacids and other orders of birds in captivity, such as Psittaciformes, Passeriformes, Columbiformes, Suliformes and Strigiformes [8–10], but is rarely observed in free-living birds [10–13].

*Sarcocystis falcatula* is endemic in the Americas, because the definitive host of this parasite are opossums of the genus *Didelphis* that is exclusive from American continents. When infected by *S*. *falcatula*, birds that are non-endemic in Americas tend to suffer from severe infections, with high mortality rates, in contrast to birds that are native from these continents. This difference is explained by interpretations of an evolutionary nature, since birds of the American continents are sympatric to the definitive hosts of *S*. *falcatula* and have evolved in the presence of this agent, which must have caused them to adapt to the infection [8,14,15].

Magellanic penguins (*Spheniscus magellanicus*) reproduce in areas of the Atlantic and Pacific oceans of South America, in Argentina, Chile and the Falkland Islands. The annual cycle of these birds is closely linked to their seasonal nature. In September, males and females return to the breeding colonies, where they begin to nest. The chicks, which are born about 40 days after the eggs are laid, take about 70 days to become independent. After the breeding season, the animals undergo their annual molting, ending their period on dry land [16,17] and begin their migration. These birds migrate to Peru and Brazil between May and September [17,18] in search of food, and are often found stranded on Brazilian beaches. The vast majority of birds that become stranded are juveniles and susceptible to the environmental and anthropogenic challenges that their first migration entails. Stranded penguins are rescued and sent to Rehabilitation Centers. Depending on their state of health, they are kept in captivity until they are ready for reintroduction into the wild, or are kept in captivity if they are considered unfit for free living [19,20].

Few reports on tissue cyst forming coccidia in penguins are available in the scientific literature, and most of them are descriptive studies of serological surveys or case reports [21–25]. The purpose of this study was to detect infections by these protozoans in Magellanic penguins rescued along the Brazilian coast by means of molecular and serological methods.

## Materials and methods

### Ethics

The capture and collection of biological samples from the birds were authorized by means of permits nos. 36250–5 and 26896–3 granted by the Biodiversity Authorization and Information System (SISBIO) of the Brazilian Institute of Environment and Renewable Natural Resources (IBAMA). This study was approved by the Ethics Committee on Animal Use (CEUA) of the Faculty of Veterinary Medicine and Animal Science—University of São Paulo (FMVZ-USP), under Protocol no. 9701041113.

### Provenance of the animals

To collect biological material from penguins, two campaigns were conducted from May to November 2014 and 2015 –the period when these birds become stranded on the Brazilian coast–both of them at the Institute for Marine Animal Research and Rehabilitation (IPRAM) in Cariacica, state of Espírito Santo, Brazil. All the stranded animals were juveniles (<4 years old). Due to the absence of stranded birds in 2014, it was decided to obtain samples from rescued birds that had died in previous years (2012 and 2013) and whose carcasses had been frozen. With this material, it was not possible to perform the mouse bioassay to isolate *T*. *gondii*; therefore, only molecular analyses were performed. The animals rescued in 2015 along the coast of the states of Espírito Santo (ES), Rio de Janeiro (RJ) and Bahia (BA) were sent to IPRAM, where blood was collected from them, as well as biological material from birds that had died during rehabilitation.

### Collection of samples

Pectoral muscle, heart and brain were harvested from carcasses frozen in previous years. The birds that died at IPRAM in 2015 were necropsied, and samples were collected from the same organs. Blood samples from the latter were collected from the jugular or femoral vein for the detection of anti-*T*. *gondii* antibodies, no less than 10 days after the bird’s arrival at IPRAM, or after the animal’s health stabilized. Blood sample were collected from some birds that survived before they were release, and during necropsy from the ones that died.

A total of 514 tissue samples were collected from 330 individuals surveyed from 2012–2015, comprising 342 samples of pectoral muscle, 86 of heart and 86 of brain. Serum were collected from 145 animals surveyed in 2015.

### Detecction of anti-*T*. *gondii* antibodies

Anti-*T*. *gondii* antibodies were detected by the modified agglutination test (MAT). Dilution of serum was made into a 96-well microplate, buffered saline solution with pH 7.2 (0.146M NaCl, 0.0026M NaH2PO4, 0.008M Na2HPO4), 45 μm membrane filtrate porosity. Serial dilutions 1: 5, 1:10 and 1:20 were made. The antigen dilution solution, composed of 2.5 mL buffered saline pH 8.95 (0.12M NaCl; 0.05M H3BO3; 0.03M NaN3; bovine serum albumin for a 0.4% solution), 35 μL of mercaptoethanol 0.2M and 50 μL Evans Blue 0.2%. Then 100 μL of antigen-stock (formaldehyde-fixed tachyzoites) was added. This mixture was homogenized and 25 μL were immediately distributed in each well of the microplate. Diluted sera were transferred to the wells of the microplate and mixed with reagent. The plate was sealed with adhesive plastic to avoid evaporation and incubated for 12 hours in an oven at 37° C. The formation of a contoured button at the bottom of the well was considered negative result; binding of the antigen and antibody forms a mesh or veil in the surface of the well, this being the positive result, as described by [26]. The cutoff point used here was 1:20 [25].

### Molecular identification

The total DNA content was extracted from tissue samples and purified using a DNeasy Blood & Tissue Kit (Qiagen, Hilden, Germany) following the manufacturer’s recommendations, except for the elution of the final product, which was done in a volume of 50 μl of the elution buffer (AE buffer). Typically, 25 to 50 mg of tissue samples were submitted to DNA extraction.

DNA samples were subjected to PCR and sequencing of PCR products with primers directed to the coding sequence of the smallest ribosomal unit (PCR-18S) for the molecular identification of organisms of the family Sarcocystidae [27]. DNA samples that tested positive for the presence of 18S sequences of the genus *Sarcocystis* were subjected to nested PCR amplification and sequencing with 18S- and 5.8S-directed primers flanking the internal transcribed spacer 1 (ITS1). The primers were JS4 [28], JS4b, CT2b and CT2c [29]. The cycling conditions were done as described elsewhere [29].

Samples in which ITS1 sequences were identified as being related *to S*. *falcatula* were subjected to PCR amplification and sequencing with SAG2, SAG3 and SAG4 (SAG–surface antigen gene) primers for multilocus differentiation among Sarcocystis species. The primers used were SAG2F1, SAG2R1, SAG3F1, SAG3R1, SAG4F2, SAG4R as previously described [30]. In these samples, investigations also focused on fragments of the cytochrome B (CYTB) encoding gene of the mitochondrial genome and on fragments of the RNA polymerase (RPOB) beta subunit-encoding gene of the apicoplast genome. The primers (CytBF, CytBR, RpoBF, RpoBR) were used as previously described [31].

The PCR products were subjected agarose gel electrophoresis and stained with Syber Safe (Eugene, OR, USA) under UV transillumination, following the manufacturer’s specifications. The PCR products subjected to nucleic acid sequencing were previously purified using ExoSap-IT (USB, Cleveland, Ohio, USA), according to the manufacturer’s specifications. The purified products were sequenced bidirectionally using a BigDye Terminator v3.1 Cycle Sequencing Kit (code 4337456, Applied Biosystems, CA, USA) and an ABI 3730 DNA Analyzer (Applied Biosystems, CA, USA).

The MEGA7 program was used to identify the nucleotides between gene sequences [32]. Phylogenetic trees were built using the Maximum Likelihood estimation (MLE) method. The evolutionary history, nucleotide substitution models, and the statistical bootstrap method to evaluate the consistency of the tree branches were inferred with the help of the MEGA7 program [32].

The ITS1 sequences of *Sarcocystis* spp. from penguins were compared with similar sequences available in GenBank using the BLAST search tool. Sequences with more than 90% coverage containing at most one degenerate site were chosen for phylogenetic reconstruction, using the above described methods.

## Results

**Fig 1:**
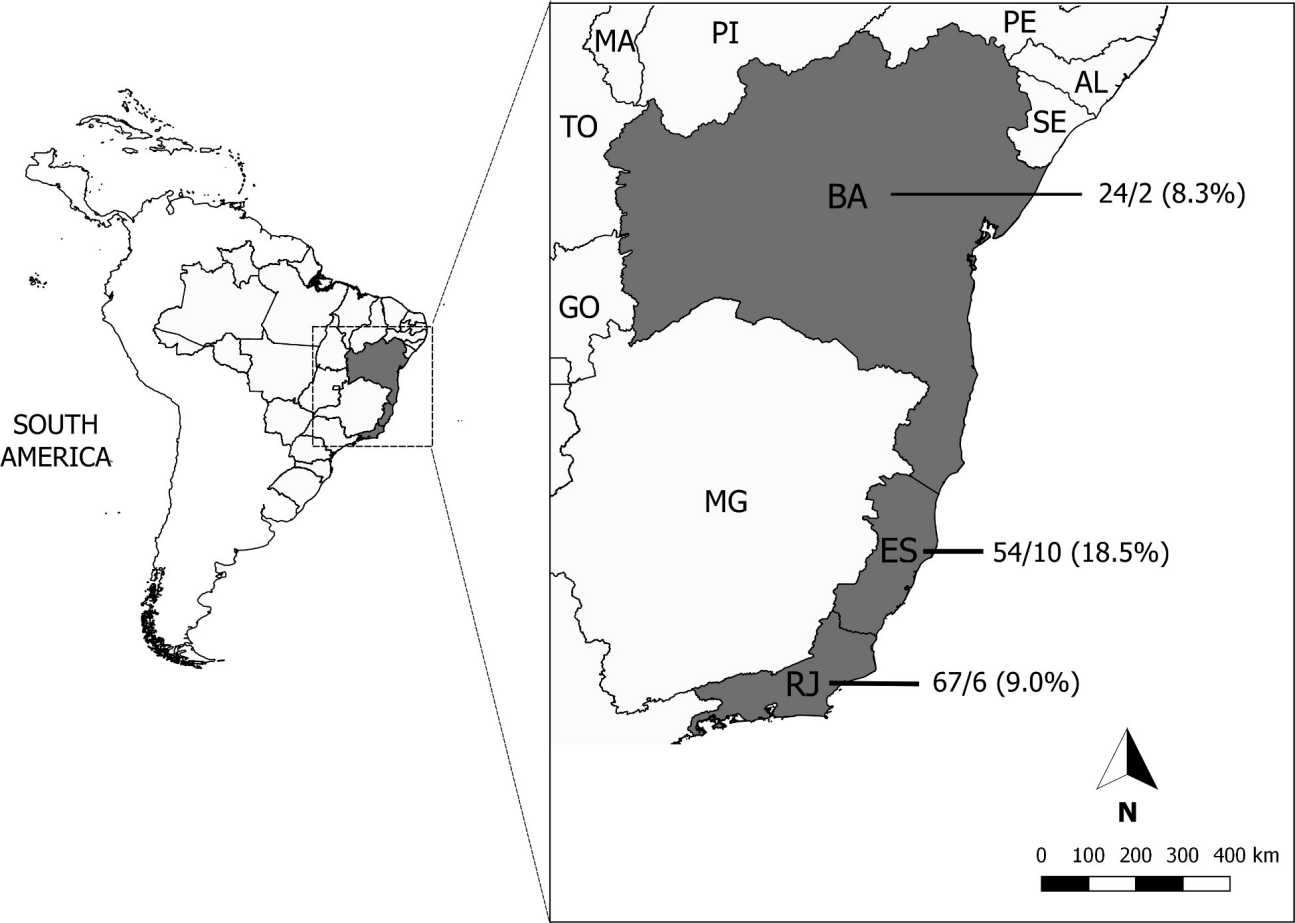
Distribution of *T*. *gondii* seroreactive animals per state. Data showed on the map is represented as follows: total number of animals surveyed per state/number of seroreactive animals (percentage of positive animals per state).

Anti-*T*. *gondii* antibodies in titers of 80, 40, 20 and <20 were found in 2, 9, 7, and 127 birds respectively. None of the seropositive animals was PCR-18S positive for either *T*. *gondii* or *Sarcocystis* spp. As for the distribution of reactive birds per state, the largest number of penguins were analyzed in the state of Rio de Janeiro and the highest percentage of animals that tested positive (18.5%) was found in the state of Espírito Santo (Fig 1).

All the samples (514) were subjected to PCR-18S, and sequences of organisms of the genus *Sarcocystis* were identified in 16 of them (3.0%). All the samples that tested PCR-18S positive were of pectoral muscles from different birds and were positive by PCRs targeted to ITS1, CYTB, RPOB, SAG2, SAG3 and SAG4. Among the PCR-18S-positive birds, 7 were sampled in 2012, 3 in 2013 and 6 in 2017. Genetic sequences from these markers were identical in all the 16 samples, and then genetic sequences of one of the samples were deposited in Genbank, identified by numbers: MG493471 (ITS1), MG493470 (CYTB), MG493472 (RPOB), MG493467 (SAG2), MG493468 (SAG3) and MG493469 (SAG4). Sequencing was performed on 988, 606, 455, 471, 373 and 308 nucleotides of ITS1, CYTB, RPOB, SAG3, SAG2 and SAG4, respectively.

**Fig 2:**
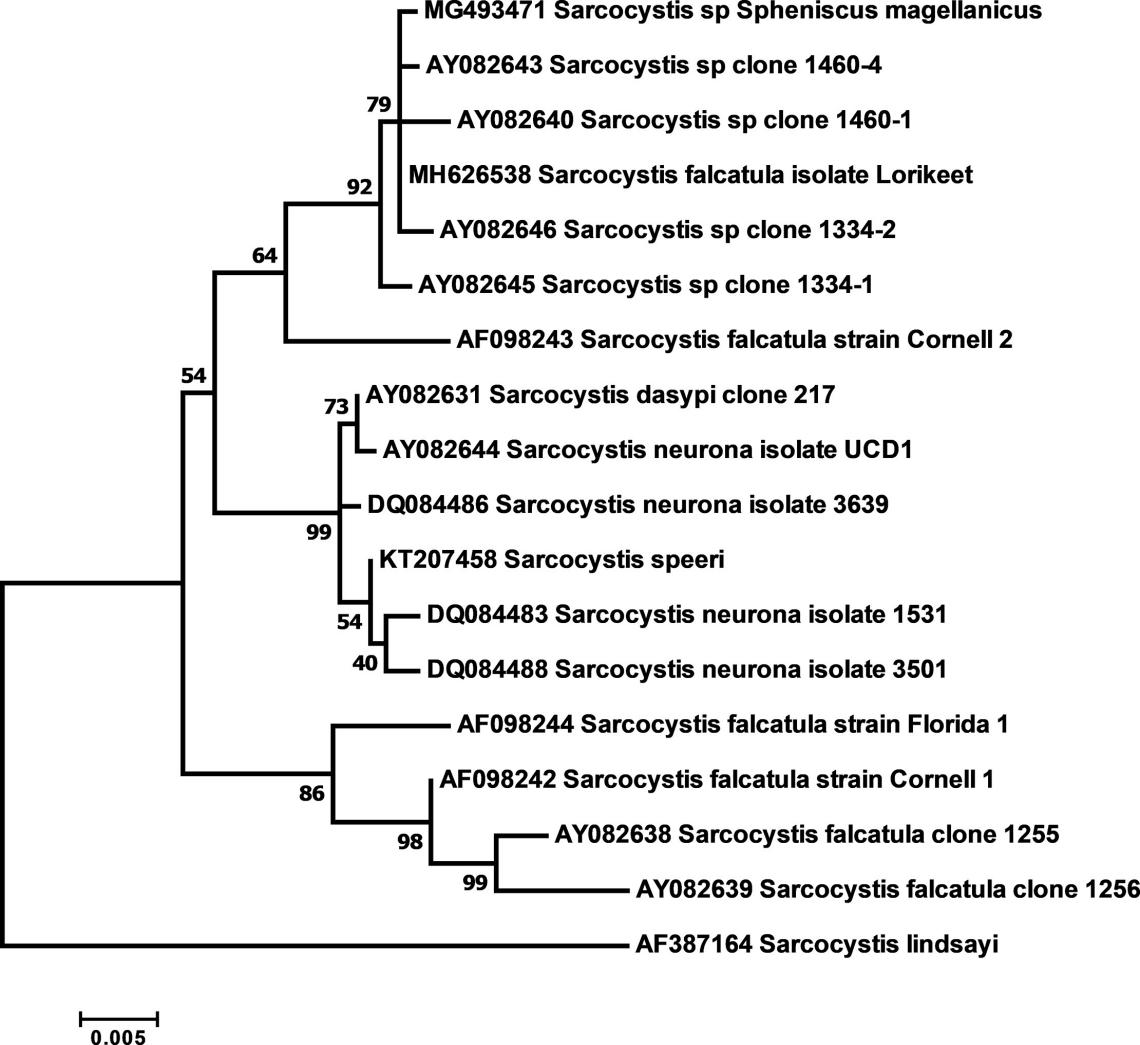
Phylogenetic analysis of Sarcocystidae related to *Sarcocystis* spp. of Magellanic penguins (*Spheniscus magellanicus*) based on internal transcribed spacer 1 (ITS1). The evolutionary history was inferred by using the maximum likelihood method based on the Hasegawa-Kishino-Yano nucleotide substitution model.

At ITS1 locus, the sequences detected in tissues of penguins showed high similarity to *Sarcocystis* spp. clones from the seabird *Morus bassanus* and the opossum *Didelphis virginiana* (AY082640, AY082641, AY082642, AY082643, AY082645, AY082646, AY082647), which in turn is closely related to organisms classified as *S*. *falcatula* and *S*. *neurona* from other hosts. This clade includes sequence from *S*. *falcatula* detected in lethal infections of Lorikeet (*Trichoglossus moluccanus*) (MH626538) [13]. A phylogenetic reconstruction with these sequences and with representatives of *S*. *falcatula* and *S*. *neurona* from Genbank shows the occurrence of three clades, well supported statistically (Fig 2). One of the clades contain sequences similar to those of *Sarcocystis* spp. of penguins another have sequences of *S*. *neurona* and a third with sequences of *S*. *falcatula*.

At ITS1 and CYTB loci, the genetic sequences obtained from penguins were identical to genotypes I and II, respectively, of *Sarcocystis* spp. identified in sporocysts of didelphid opossums by VALADAS et al [30] in Brazil. For these markers, the *Sarcocystis* spp. sequences from penguins were also identical to isolate 59-2016-RS-BR of *S*. *falcatula* from naturally infected *Phimosus infuscatus* (bare-faced ibis) in Brazil (KX2650186 and KX265018) [11] and identical to *Sarcocystis* spp. isolated in parakeets experimentally infected with sporocysts of *Sarcocystis* spp. [33,34] from Brazilian opossums.

For RPOB, the sequences of *S*. *magellanicus* is high similar to sequences from bird parasites *S*. *falcatula* isolate SF1, *S*. *lindsayi* and *S*. *falcatula* isolated from bare-faced ibis. The sequences of parasites from birds differ from each other in 1 to 2 nucleotides, while *S*. *neurona* differs from the *Sarcocystis* sequences of the avian clade in 3 to 4 nucleotides.

The SAG sequences obtained from 16 samples were 100% identical to the sequences of homologous alleles of *Sarcocystis* spp. identified as sporocysts from didelphid opossums in Brazil and classified as types III (JN185358), III (JN185386) and XI (JN185800), as described by Monteiro et al [35] for SAG2, SAG3 and SAG4, respectively.

In SAG2, the penguin samples exhibited 99.5 and 97.3% identity with *S*. *falcatula* and *S*. *neurona*, respectively. For SAG3, these values were 96.0% and 90.9%, respectively. For SAG4, the values of identity with *S*. *falcatula* and *S*. *neurona* were 92.5 and 91.6%, respectively. The following sequences available in Genbank were used for these comparisons: GQ851952 and GQ851953 (for SAG2 from *S*. *neurona* and *S*. *falcatula*, respectively), GQ851954 and GQ851956 (for SAG3 from *S*. *neurona* and *S*. *falcatula*, respectively), and GQ851958 and GQ851959 (for SAG4 from *S*. *neurona* and *S*. *falcatula*, respectively).

Data indicating identity of genetic sequences of penguin *Sarcocystis* with homologous from Genbank are in S1 Table.

## Discussion

This is the first report on molecular detection of infection by *S*. *falcatula*-related organisms and the first report of seropositivity for *T*. *gondii* in free-living Magellanic penguins in Brazil.

The MAT used in this study is considered the most sensitive and specific test for the detection of anti-*T*. *gondii* antibodies in animals [2]. Although the test has not been validated for the detection of *T*. *gondii* in penguins, it has been widely used in other bird species, aquatic or not [2,25] and the titer of 20 is significantly higher than the one standardized for chickens by Dubey et al [36], who isolated *T*. *gondii* in animals with titers of 5 in MAT.

In the scientific literature, the data on the detection of anti-*T*. *gondii* antibodies in penguins is scanty, and refers mainly to free-living animals, which are the target of this study. Using MAT, Deem et al [24] investigated anti-*T*. *gondii* antibodies in 298 free-living Galapagos penguins (*Spheniscus mendiculus*) on the island of Fernandina, in Ecuador. These authors used a cutoff point of 1:50, which is higher than the 1:20 cutoff used here, and found an occurrence of 2.8%.

In a study also involving Magellanic penguins, albeit living in captivity, Gennari et al [25] used a cutoff point of 1:20 in MAT and found that 28 of the 100 penguins (28%) they analyzed tested positive for anti-*T*. *gondii* antibodies, which is more than double the rate found here, indicating that living in captivity may facilitate exposure to *T*. *gondii*.

Although seropositivity for *T*. *gondii* was detected in this study, this coccidian parasite was neither isolated in the mouse bioassay, nor PCR-detected in the collected tissues. A possible explanation for this is that infections by coccidian protozoa do not always produce sufficient tissue cysts to enable mouse infection or PCR detection. A very small amount of tissue is processed in DNA extraction kit used in this study, thus the odds of there being a tissue cyst of *T*. *gondii* in that portion of tissue are low. According to Dubey [37], muscle cysts in large animals usually correspond to an average of one cyst per 25–250 grams, which is considered low. In addition, false positives on MAT should not be discarded due to unknown serum related factors that may interfere with the test. False positives on MAT have already been reported for Arctic marine wildlife due to the high lipid levels of blood. Seropositivity for *T*. *gondii* was not associated to the presence of DNA of *Sarcocystis* spp. in tissues either, thus MAT seropositivity was not due to Sarcocystis infection.

Regarding *Sarcocystis* infection, identical sequences for each marker (ITS1, SAG2, SAG3, SAG4, CYTB and RPOB) were found in all the samples (n = 16), indicating that the 16 birds were all infected by the same *Sarcocystis* species. The multilocus analysis of the muscle tissue samples of *S*. *magellanicus* allows us to conclude that these birds were infected by a parasite closely related to *S*. *falcatula*. The ITS1 and CYTB fragments from these samples were identical to the homologous sequences from *Sarcocystis falcatula* of bare-faced ibis found on the coast of the state of Rio Grande do Sul, Brazil [11]. ITS1 and CYTB alleles of *Sarcocystis* spp. of *S*. *magellanicus* were also identical to homologous fragments from highly parakeet pathogenic *Sarcocystis* spp. [33,34], indicating that all Sarcocystidae described in these studies are organisms that belong to the same species.

The SAG2, SAG3 and SAG4 alleles of *Sarcocystis* spp. of *S*. *magellanicus* are identical to homologous alleles obtained from sporocysts of *Sarcocystis* spp. passed with the feces of didelphid opossums in Brazil [30,35]. Together, these authors found 10 variants for SAG2, 15 for SAG3 and 11 for SAG4 in about 50 samples of sporocysts, and suggested that *Sarcocystis* transmitted by didelphids could produce parasites with different combinations of SAG alleles, which would confer different phenotypic traits to the parasite, such as the host spectrum and pathogenicity. However, in spite of the intense abundance of SAG alleles found in Brazil, all the samples detected in this study were identical to each other for each allele.

Furthermore, the combination of SAG2, SAG3, and SAG4 in these samples differs from the combination found in *S*. *falcatula* isolate SF1, which, for these loci, has allele types X, XV and XII, respectively. The combination of SAGs of *Sarcocystis* spp. of *S*. *magellanicus* also differs from those found in the experimental infection studies by Gondim et al [33] and Cesar et al [34] and also differs from that found in bare-faced ibis [12]. Thus, the results reported here seem to corroborate the hypothesis formulated by Monteiro et al [35] and Valadas et al [30], since the agent found in penguin tissues should be a variant of a species of the genus *Sarcocystis* most closely related to *S*. *falcatula* with a particular combination of SAG alleles.

Morphological studies would be essential mainly to show mature sarcocysts proving that the penguins would be HI of the parasite and helping make the species designation. Unfortunately, the tissues were preserved for isolation of *T*. *gondii* by bioassay, and therefore were not preserved for histological studies. On the other hand, even with the morphological data, it would not be possible to close the question that these *Sarcocystis* would be from a new species. In fact, *S*. *falcatula* and the *Sarcocystis* detected here have important differences from the point of view of molecular phylogenies, but on the other hand, this can be explained by the geographic isolation between these two organisms. It is possible that both agents, in spite of their genetic distance, can exchange genes in an eventual gamogony within definitive host. If this is possible, both organisms should be classified as belonging to the same species, as they would fit into the biological concept of species. Regarding infectivity for HI, *S*. *falcatula* and the parasites detected in the studies of Gondim et al. [33] and Cesar et al. [34] appear to behave identically, but from the phylogenetic point of view, both have important differences. As pointed above, *Sarcocystis* from the studies of Gondim et al. [33] and Cesar et al. [34] and the parasites detected in Magellanicus penguins are presumably to belong to the same species. In the United States, non-American psittacines of the species *Trichoglossus moluccanus* were diagnosed with lethal sarcocistosis [13] and ITS1 sequences of the parasites were close related to the homologous from *Sarcocystis* found in Magellanicus penguins. In this work, the authors classified the coccidian in the species *S*. *falcatula*. Thus, if *Sarcocystis* spp. from penguins and *S*. *falcatula* are distinct entities remains to be elucidated.

Numerous outbreaks of acute sarcosporidiosis by *S*. *falcatula* have been reported in birds of the orders Passeriformes, Psittacids, Columbiformes, Strigiformes and Falconiformes living in captivity in the Americas [8,9,38–41]. *S*. *falcatula* can reportedly cause severe respiratory disease in captive parrots [8,9,40], but there are only two reports of it in free-living birds [10,11], which were diagnosed using methodologies similar to those employed here. In this study, all the penguins were undergoing rehabilitation and died due to natural causes. Given that parasite genetic sequences were detected in samples of muscle tissue, it can be inferred that bradyzoites of *Sarcocystis* were detected, which are structures characteristic of the chronic phase of the infection, and hence, unlikely that the parasite was causing disease in the host.

The *Sarcocystis* derived sequences were from the pectoral muscle of the penguins and it is impossible to determine when these birds were infected. Luznar et al [42] infected *Molothrus ater* (Brown-headed cowbird), a migratory aquatic bird, and observed that *S*. *falcatula* sarcocysts remained viable for at least 40 weeks post infection in the natural intermediate host, indicating long-lasting viability in the cysts of hosts. Moreover, it is known that opossums infected experimentally with sarcocysts of *S*. *falcatula* from tissues of *M*. *ater* eliminated sporocysts until they were euthanized, 200 days after infection [43], implying the transmission capacity of *S*. *falcatula* by opossums. This information raises the difficult question to answer how and when the birds in this study became infected, assuming that didelphid opossums are definitive host of the *Sarcocystis* found in penguins, which can be inferred from their similarity with *Sarcocystis* detected from opossums in other studies. Thus, if penguins were infected in the sea or in land remains to be confirmed.

Regarding *T*. *gondii* infection, this coccidian oocysts can sporulate and survive in seawater for several months [44–46]. Marine mammals of different groups, cetaceans, pinnipeds and sirenians, as well as waterfowl, can become infected by consuming water containing oocysts. Cole et al [47] suggested that *T*. *gondii* oocysts, which are present in cat feces, can enter the marine environment and contaminate the waters and several invertebrates that can act as transport hosts to mammals and seabirds. Lindsay et al [48] succeeded in infecting mice fed with oysters (*Crassostrea virginica*) infected experimentally with this coccidian. The main diet of Magellanic penguins consists of fish such as sardines, mackerel, anchovies and spotted goatfish, the most frequent of which are sardines, but they also feed on small marine filter-feeding crustaceans such as *Euphausia superba*, known as Antarctic Krill, which, together with oysters, may play an important role in the *T*. *gondii* transmission [48] and possibly other sarcocystids. Nevertheless, transmission of cyst forming coccidians in the local were the penguins are allocated could not be ruled out. Thus, more detailed studies are needed focusing on the way of transmission of both agents investigated here.

## Supporting information

S1 Table Distance from GenBank nucleotide sequences with homologous from *Sarcocystis* of *Spheniscus magellanicus*. (DOCX)
