# Correction: Occurrence of tissue cyst forming coccidia in Magellanic penguins (*Spheniscus magellanicus*) rescued on the coast of Brazil

**DOI:** 10.1371/journal.pone.0212467

**Published:** 2019-02-11

**Authors:** Igor Cunha Lima Acosta, Rodrigo Martins Soares, Luis Felipe Silva Pereira Mayorga, Bruna Farias Alves, Herbert Sousa Soares, Solange Maria Gennari

In the Funding section, the scholarship number from the funder São Paulo State Research Foundation is listed incorrectly. The correct grant number is: FAPESP: 2018 / 03193–8.

## References

[1] Acosta ICL, Soares RM, Mayorga LFSP, Alves BF, Soares HS, Gennari SM (2018) Occurrence of tissue cyst forming coccidia in Magellanic penguins (*Spheniscus magellanicus*) rescued on the coast of Brazil. PLoS ONE 13(12): e0209007 10.1371/journal.pone.0209007 30562391 PMC6298673

