# Supplementary material for: Occurrence of tissue cyst forming coccidia in Magellanic penguins (Spheniscus magellanicus) rescued on the coast of Brazil
Source: PLoS One. 2018 Dec 18;13(12):e0209007. doi: 10.1371/journal.pone.0209007 (PMC6298673; doi:10.1371/journal.pone.0209007)
Supplement: S1 Table — (DOCX) [file pone.0209007.s001.docx]

**S1 Table. Distance from GenBank nucleotide sequences with homologous from *Sarcocystis* of *Spheniscus magellanicus.***

| Acession number | Species | Locus | Strain/clone | Origin | Distance with homologous sequences from Sarcocystis of Spheniscus magellanicus: MG493467 to MG 493472 (SAG2, SAG3, SAG4, CYTB, ITS1, RPOB) | | | |
| --- | --- | --- | --- | --- | --- | --- | --- | --- |
|  |  |  |  |  | *p*-distance | N° of nucleotide differences | N° gaps | N° positions aligned |
| MH626538 | *Sarcocystis falcatula* | ITS1 | Lorikeet ID #205850 | *Trichoglossus moluccanus* | 0.001 | 1 | 8 | 988 |
| AY082640 | *Sarcocystis sp* | ITS1 | clone 1460-1 | *Morus bassanus* | 0.0041 | 4 | 12 | 988 |
| AY082643 | *Sarcocystis sp* | ITS1 | clone 1460-4 | *Morus bassanus* | 0.002 | 2 | 12 | 988 |
| AY082645 | *Sarcocystis sp* | ITS1 | clone 1334-1 | *Didelphis virginiana* | 0.0041 | 4 | 12 | 988 |
| AY082646 | *Sarcocystis sp* | ITS1 | clone 1334-2 | *Didelphis virginiana* | 0.0031 | 3 | 12 | 988 |
| KT207458 | *Sarcocystis speeri* | ITS1 | #opossum 8157 | *Didelphis albiventris* | 0.0184 | 18 | 15 | 988 |
| AY082631 | *Sarcocystis dasypi* | ITS1 | clone 217 | *Dasypus novemcinctus* | 0.0194 | 19 | 15 | 988 |
| AY082644 | *Sarcocystis neurona* | ITS1 | isolate UCD1 | *Equus caballus* | 0.0204 | 20 | 15 | 988 |
| DQ084486 | *Sarcocystis neurona* | ITS1 | isolate 3639 | *Enhydra lutris* | 0.0196 | 19 | 15 | 976 |
| DQ084483 | *Sarcocystis neurona* | ITS1 | isolate 1531 | *Phoca vitulina* | 0.0215 | 21 | 15 | 988 |
| DQ084488 | *Sarcocystis neurona* | ITS1 | isolate 3501 | *Enhydra lutris* | 0.0217 | 21 | 15 | 976 |
| AF098243 | *Sarcocystis falcatula* | ITS1 | strain Cornell 2 | *Quiscalus mexicanus* | 0.0184 | 18 | 8 | 988 |
| AF098244 | *Sarcocystis falcatula* | ITS1 | Strain Florida 1 | *Quiscalus quiscula* | 0.0277 | 27 | 15 | 988 |
| AF098242 | *Sarcocystis falcatula* | ITS1 | strain Cornell 1 | *Quiscalus quiscula* | 0.0287 | 28 | 15 | 988 |
| AY082638 | *Sarcocystis falcatula* | ITS1 | clone 1255 | *Molothrus ater* | 0.0338 | 33 | 16 | 988 |
| AY082639 | *Sarcocystis falcatula* | ITS1 | clone 1256 | *Molothrus ater* | 0.0389 | 38 | 16 | 988 |
| AF387164 | *Sarcocystis lindsayi* | ITS1 |  | *Didelphis albiventris* | 0.0597 | 58 | 27 | 988 |
| KX265016 | *Sarcocystis falcatula* | ITS1 | isolate 59-2016-RS-BR | *Phimosus infuscatus* | 0 | 0 | 0 | 290 |
| KP871733 | *Sarcocystis falcatula* | RPOB | strain Florida 1 (SF1) | *Didelphis virginiana* | 0.0023 | 1 | 0 | 440 |
| KX265017 | *Sarcocystis falcatula* | RPOB | isolate 59-2016-RS-BR | *Phimosus infuscatus* | 0.0045 | 2 | 0 | 440 |
| AY164997 | *Sarcocystis lindsayi* | RPOB |  | *Didelphis albiventris* | 0.0044 | 2 | 0 | 440 |
| KP871732 | *Sarcocystis neurona* | RPOB | 138 | *Didelphis virginiana* | 0.0091 | 4 | 0 | 440 |
| JN185358 | *Sarcocystis sp* | SAG2 | isolate G02 | *Didelphis aurita* | 0 | 0 | 0 | 279 |
| GQ851952 | *Sarcocystis neurona* | SAG2 | strain 3106 | *Enhydra lutris* | 0.0268 | 10 | 0 | 373 |
| GQ851953 | *Sarcocystis falcatula* | SAG2 | strain North american | unknown | 0.0054 | 2 | 0 | 373 |
| JN185386 | *Sarcocystis sp* | SAG3 | isolate G17 | *Didelphis aurita* | 0 | 0 | 0 | 415 |
| GQ851954 | *Sarcocystis neurona* | SAG3 | strain 3106 | *Enhydra lutris* | 0.0913 | 43 | 94 | 471 |
| GQ851956 | *Sarcocystis falcatula* | SAG3 | strain North american | unknown | 0.0403 | 19 | 6 | 471 |
| JN185400 | *Sarcocystis sp* | SAG4 | isolate G29 | *Didelphis aurita* | 0 | 0 | 0 | 275 |
| GQ851958 | *Sarcocystis neurona* | SAG4 | strain 3639 | *Enhydra lutris* | 0.0844 | 26 | 0 | 308 |
| GQ851959 | *Sarcocystis falcatula* | SAG4 | strain North american | unknown | 0.0747 | 23 | 0 | 308 |
| KP871704 | *Sarcocystis falcatula* | CYTB | strain Florida 1 (SF1) | *Didelphis virginiana* | 0 | 0 | 0 | 580 |
| KP871703 | *Sarcocystis neurona* | CYTB | 138 | *Didelphis virginiana* | 0.0017 | 1 | 0 | 580 |
| KX265018 | *Sarcocystis falcatula* | CYTB | isolate 59-2016-RS-BR | *Phimosus infuscatus* | 0 | 0 | 0 | 580 |
